# Value of biplane transrectal ultrasonography plus micro-flow imaging in preoperative T staging and rectal cancer diagnosis in combination with CEA/CA199 and MRI

**DOI:** 10.1186/s12885-023-11370-8

**Published:** 2023-09-12

**Authors:** Qin Xia, Wei Cheng, Jie Bi, An-Ping Ren, Xiao Chen, Tao Li

**Affiliations:** Department of Ultrasound, Daping Hospital, Army Medical University, Chongqing, 400000 China

**Keywords:** Biplane TRUS, MFI, Rectal Cancer, Tumor staging, CEA, CA199, MRI, Combined

## Abstract

**Background:**

Rectal cancer is one of the most common malignant tumors and has a high incidence rate and fatality rate. Accurate preoperative T staging of rectal cancer is critical for the selection of appropriate rectal cancer treatment. Various pre-operative imaging methods are available, and the identification of the most accurate method for clinical use is essential for patient care. We investigated the value of biplane transrectal ultrasonography (TRUS) combined with MFI in preoperative staging of rectal cancer and explored the value of combining TRUS plus MFI with CEA/CA199 and MRI.

**Methods:**

A total of 87 patients from Daping Hospital with rectal cancer who underwent TRUS examination plus MFI were included. Grades of MFI were determined by Alder classification. Among the total patients, 64 underwent MRI and serum CEA/CA199 tests additionally within one week of TRUS. Pathological results were used as the gold standard for cancer staging. Concordance rates between TRUS, MRI, and CEA/CA199 for tumors at different stages were compared.

**Results:**

There were no significant differences between the Alder classification and pathological T staging. The concordance rate of TRUS and MFI for rectal cancer T staging was 72.4% (*K =* 0.615, *p* < 0.001). Serum CEA and CA199 levels were significantly different in tumors at different stages and increased progressively by pathological stage (*p <* 0.001); the accuracy rate was 71.88% (*K =* 0.599, *p <* 0.001), while that of MRI was 51.56% (*K =* 0.303, *p <* 0.001), indicating that TRUS had higher consistency in the preoperative T staging of rectal cancer. The combination of TRUS, MRI, and CEA/CA199 yielded an accuracy rate of 90.6%, which was higher than that of any method alone.

**Conclusions:**

Preoperative T staging of rectal cancer from biplane TRUS plus MFI was highly consistent with postoperative pathological T staging. TRUS combined with MRI and serum CEA/CA199 had a greater value in the diagnosis of rectal cancer and a higher diagnostic rate than any examination alone.

## Introduction

Rectal cancer is one of the most common malignant tumors. The incidence rate of rectal cancer ranks third and the fatality rate ranks second among malignant tumors worldwide [1, 2]. As reported by the World Health Organization International Cancer Research Center, there were 1.93 million new colorectal cancer cases in 2020, and 0.94 million patients succumbed to disease [3]. The depth of rectal cancer infiltration is an important prognostic factor affecting the choice of clinical treatment. The National Comprehensive Cancer Network (NCCN) Clinical Practice Guidelines for rectal cancer indicate that patients with early stage rectal cancer (T1–T2) can be treated by local excision surgery, while patients with advanced T stage (T3–T4) are advised to undergo neoadjuvant chemoradiation therapy before surgery to improve resectability and reduce the postoperative local recurrence rate [4]. Thus, preoperative T staging plays an essential role in rectal cancer treatment.

Transrectal ultrasonography (TRUS) and magnetic resonance imaging (MRI) are both widely used for preoperative T staging of rectal cancer [5]. MRI is a diagnostic technique that can visualize the entire rectal wall and mesenteric fascia and obtain an overall view of the mass [6–8]. TRUS is an ultrasound technique that can distinguish between different layers of rectal wall infiltration, especially in early stage tumors [9]. A new advanced ultrasound modality, termed micro-flow imaging (MFI), is more sensitive and specific in detecting low blood flow signals within the lesion compared with color Doppler [10].

Tumor markers are molecules that play an important role in the germination and development of tumors [11]. Carcinoembryonic antigen (CEA) and carbohydrate antigen 19 − 9 (CA19-9) are commonly used as biomarkers for the diagnosis and follow-up of colorectal cancer [12]. CEA is a high-molecular-weight glycoprotein produced by colon cells derived from human endodermal epithelial tissue that was first identified in the tissue extracts of rectal cancer patients. CA199 is a high-molecular-weight low glycolipid that is present in the form of sialomucin in the serum and is widely distributed on the cytomembrane and currently is used for the diagnosis of rectal cancer [13] However, the poor sensitivity and specificity of CEA and CA199 limit the use of only these biomarkers in the diagnosis of rectal cancer [14].

In this study, we investigated the value of biplane TRUS combined with MFI in preoperative staging for rectal cancer and examined effect of combination with laboratory examination of CEA/CA199 and MRI. We aimed to identify an approach to obtain accurate information on preoperative T staging of rectal cancer to formulate better surgical methods and improve the survival of patients.

## Materials and methods

### Subjects

**Fig. 1:**
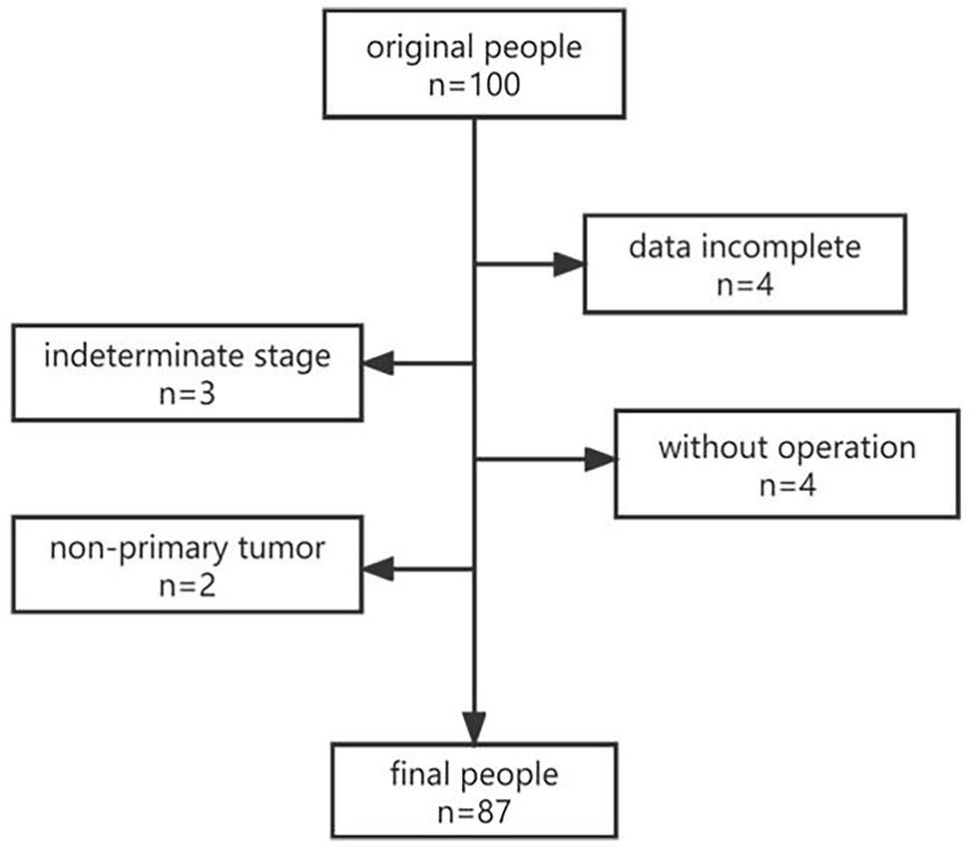
Exclusion process

In this retrospective study, 96 consecutive patients with pathologically proven rectal cancer who underwent TRUS examination at Daping Hospital, Army Medical University between January 2021 and May 2022 were evaluated. The inclusion criteria were as follows [15]: (i) the lower edge of tumor was < 15 cm from the anal verge; (ii) the patient underwent surgical treatment, allowing definitive rectal cancer staging through pathologic diagnosis, which is regarded as the gold standard; (iii) all lesions were primary lesions; and (iv) written informed consent was obtained from the patient before the examination. The following patients were excluded: (i) patients with distant metastasis; (ii) patients with missing data or unsatisfactory imaging findings; and (iii) patients with severe mental disease or who were unable to cooperate with the examinations. After applying these criteria, 87 patients were included in the study [Fig. 1]. The patient group included 51 males and 32 females, with a mean age (± standard deviation [SD]) of 59.5 ± 13.1 years (range 25–85). Among the 87 patients, 64 patients additionally underwent CA199/CEA and MRI evaluation within one week of TRUS.

### Equipment and methods

#### Ultrasound system

Ultrasonography was performed using the SonoScape P60 ultrasound system. The intracavity probe was a BCL10-5 probe, with a frequency of 3–9 MHz. The patients received an enema 2 h before the examination. During the examination, the patients were maintained in the left lateral decubitus position with their legs bent close to the anterior abdominal wall. A digital rectal examination was first conducted to preliminarily assess the tumor size and location, excluding severe stenosis of the intestinal cavity. Next, 50–100 ml normal saline was injected into the rectal cavity. A small couplant was applied to the surface of the probe and a rubber condom was placed on it; the couplant was daubed on its surface. A biplane probe with a length of approximately 120 mm was inserted into the anus slowly to ensure that the probe crossed the upper edge of the tumor. The probe was turned slowly during the examination until the rectal mass and its surrounding tissue structure were clearly displayed. The area was then scanned to record all features of the mass, including location, size, echogenicity, margin, depth of invasion, distance from the anal verge, blood flow, status of the perirectal lymph nodes, and involvement of adjacent tissues. Tumor blood flow was displayed using color Doppler ultrasound, and the arterial blood flow resistance index (RI) was measured and recorded. Initial T staging was performed during the examination from two-dimensional ultrasound and color Doppler ultrasound images.

The probe was then switched to MFI mode; the sampling box was adjusted to clearly cover the lesions and ensure it was greater than 1/2 times of the screen. The color gain of the two blood flow imaging techniques was adjusted to be as high as possible, while the ruler was adjusted to be as low as possible with no color interference. MFI assessment is strongly dependent on the operator. During the examination, any extra pressure from the microvessels was prevented so as not to influence the results. The microcirculatory perfusion of the tumor was then observed, and the most satisfactory pictures with the most abundant color flow and clearest image quality were obtained; these images were used for microvascular classification using the Adler semi-quantitative method [16].

#### MRI examination

A Siemens Magnetom Verio 3.0T (A Tim System, Magnetom, Verio) and GE Signa HDx 1.5T (GE Medical System, Milwaukee, WI, USA) magnetic resonance scanner equipped with an eight-channel phased-array abdominal coil was used. The patient was asked to remove jewelry and metal items prior to examination. The patient was placed in the supine position, and the scanning scope was moved from the diaphragm to the inferior symphysis pubis. Horizontal scanning was performed as follows: axial (perpendicular to the rectal axis), sagittal, and coronal position scanned by T2WI fast spin-echo (FSE) sequence, with a visual field (FOV) of 384 × 384 cm, layer thickness = 3 mm, layer spacing = 1 mm, repetition time (TR) = 5700–6861 ms, echo time (TE) = 92 ms. Axial T1WI used single-time excitation spin-echo plane imaging, with FOV = 320 × 320 cm, layer thickness = 5 mm, layer spacing = 1 mm, TR = 420–650 ms, TE = 10 ms. Diffusion-weighted imaging (DWI) was performed using single-shot excitation rotary echo planar imaging (FOV, 176 × 256 cm; layer thickness, 5 mm; layer spacing, 1 mm, TR = 8900 ms, TE = 75 ms. For enhanced scanning, Gd-DTPA 0.2 mmol/kg was administered as the contrast agent via the antebrachial vein at a rate of 2.0–3.0 mL/s.

The MRI images were reviewed by several experienced senior radiologists to determine the T stage of the tumor. The MRI T-staging for each patient was determined following the AJCC/TNM classification [17]. The time between receiving TRUS and MRI examination was one week or less.

#### CEA/CA199 examination

Serum CEA and CA199 were determined using the Elecsys electrochemiluminescence immunoassay (Roche Diagnostics); all assays were performed in one laboratory. The reference normal range of CEA was 0–5 ng/ml and that of CA199 was 0–37 U/ml. The time between measuring serum concentrations of CEA and CA199 and TRUS and MRI examination was one week or less.

### Staging criteria

**Fig. 2:**
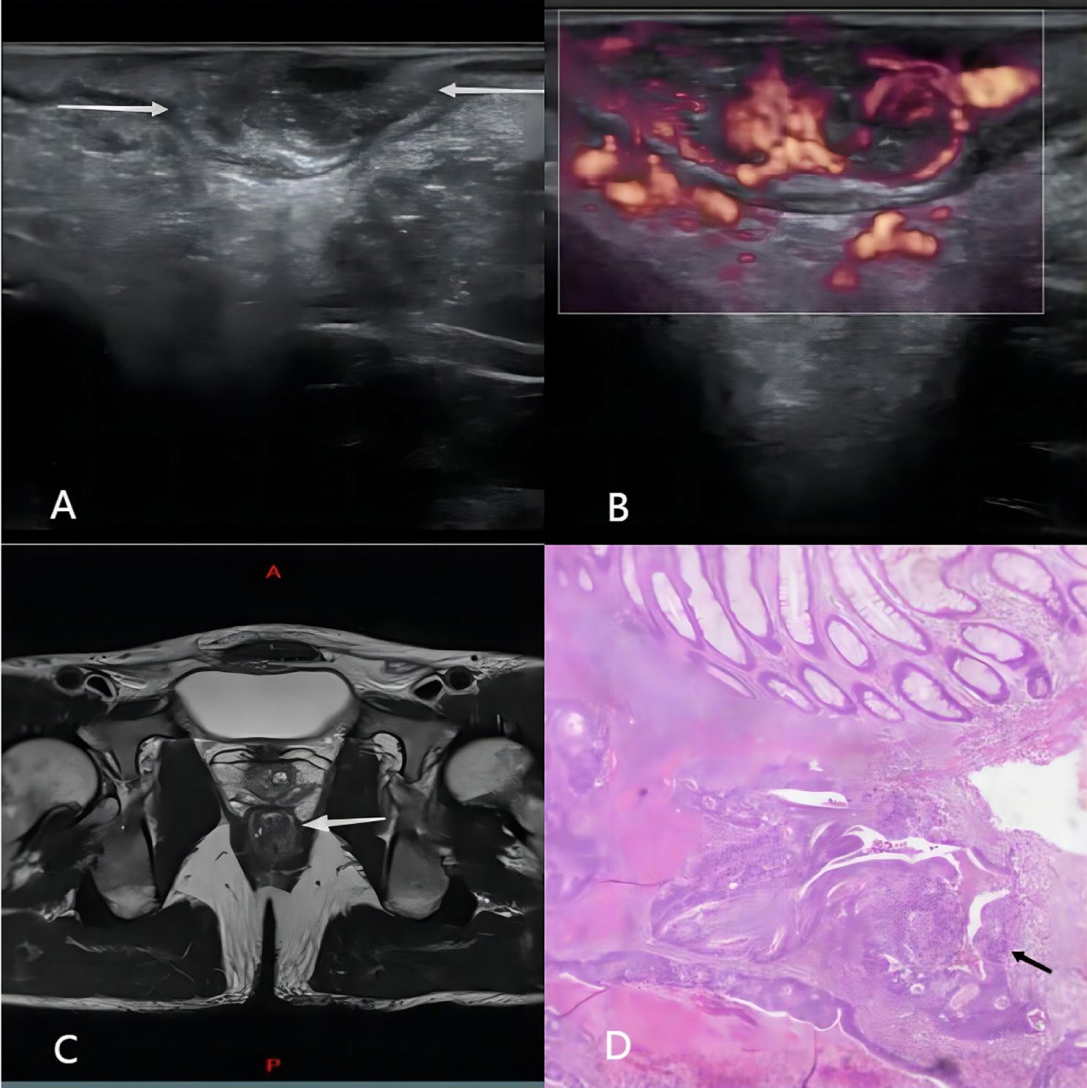
Images of a 47-year-old man with a T1 stage rectal tumor. (**A**) Two-dimensional endorectal biplane ultrasound of the line array revealed a hypoechoic mass on the rectal wall that was confined to the submucosa with a continuous hyper-echoic submucosa (arrow); the diagnosis was T1 rectal cancer. (**B**) An MFI mode image revealed that the microblood flow signals of the mass were abundant; there were more than four blood flow streams and spread to more than 50% of the maximum section of the tumor. (**C**) Magnetic resonance imaging revealing thickening of the wall of the lower rectum under peritoneal reflection (arrow); this was diagnosed as T1 rectal cancer. (**D**) The pathological diagnosis was moderately differentiated adenocarcinoma stage T1 as ulcerative type, and the cancerous tissue adjacent to the submucosa did not exceed the submucosa (arrow)

**Fig. 3:**
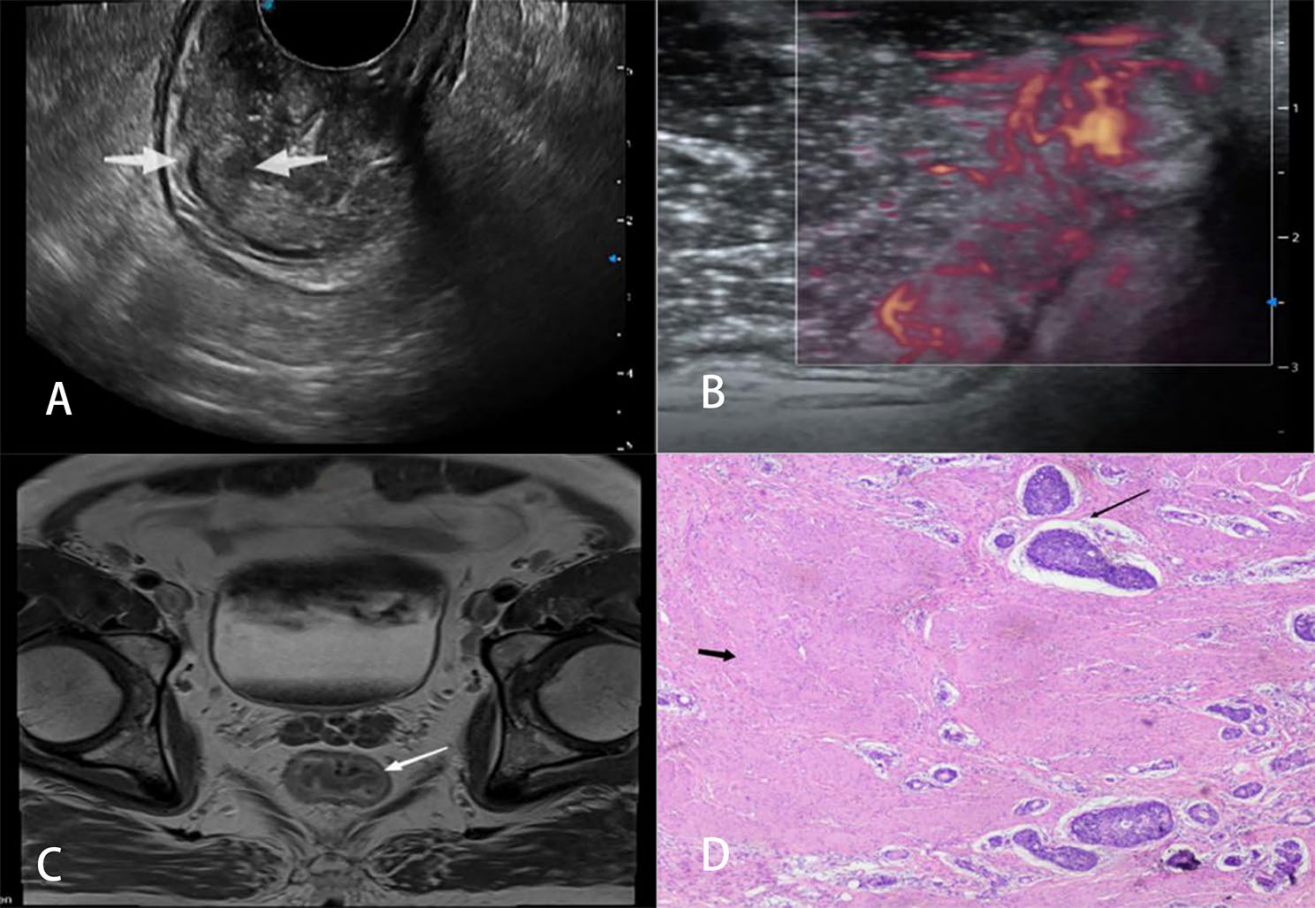
Images of a 70-year-old man with a T2 stage rectal tumor. (**A**) Two-dimensional endorectal biplane ultrasound of the convex array revealed a hypoechoic mass on the rectal wall that penetrated the submucosa and was localized in the muscularis propria (arrows); the diagnosis was T2 rectal cancer. (**B**) An MFI mode image revealed that the microblood flow signals of the mass were abundant; there were more than four blood flow streams that spread to more than 50% of the maximum section of the tumor. (**C**) Magnetic resonance imaging revealed thickening of the wall of the lower rectum under peritoneal reflection (arrow); this was diagnosed as T2 rectal cancer. (**D**) The pathological diagnosis was moderately differentiated adenocarcinoma stage T2 as ulcerative type; the cancerous tissue (long arrow) had invaded the superficial muscularis propria (short arrow) and was confined to the muscularis propria (the entire view is filled with the muscularis propria)

**Fig. 4:**
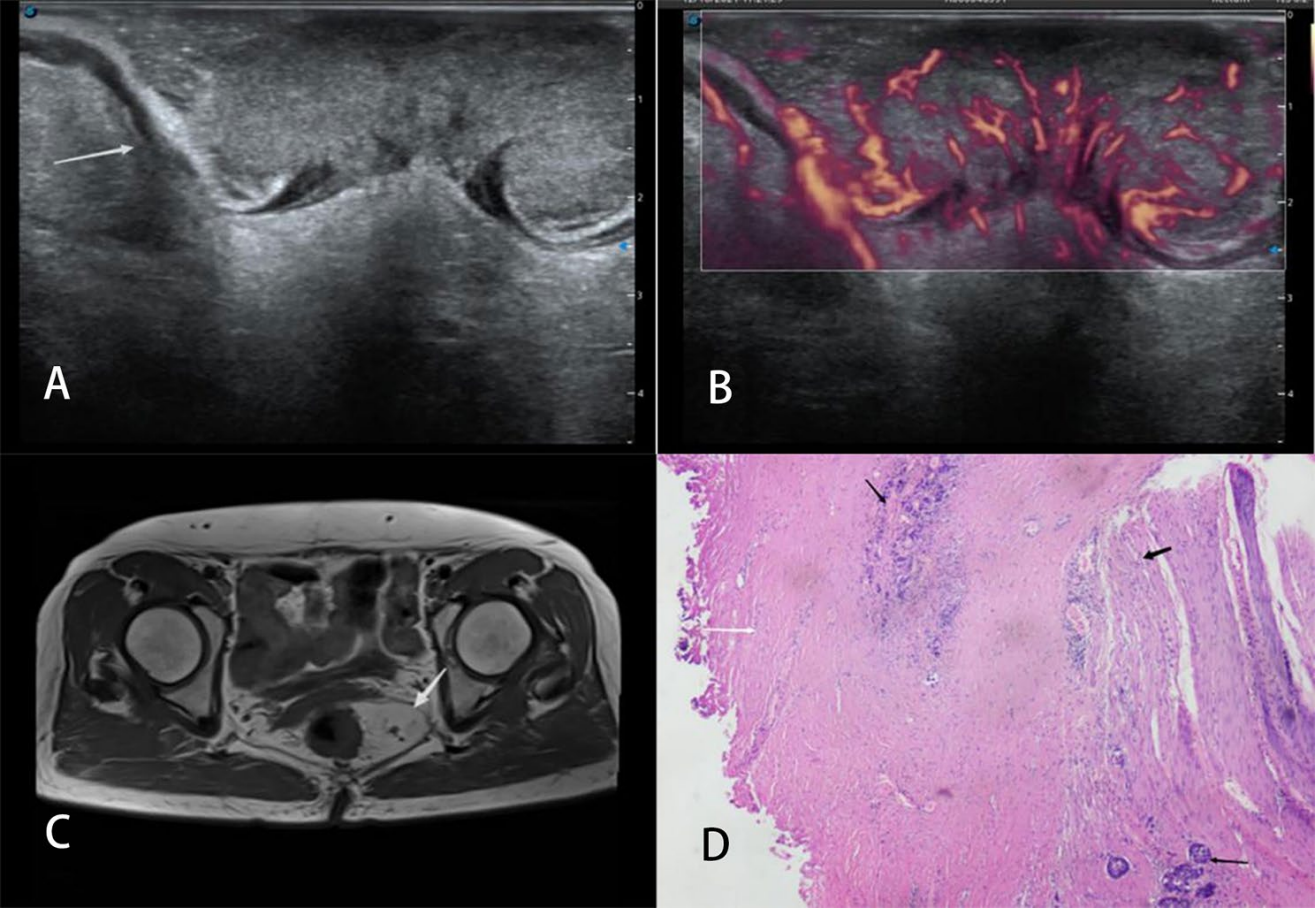
Images of a 68-year-old woman with a T3 stage rectal tumor. (**A**) Two-dimensional endorectal biplane ultrasound of the line array revealed a hypoechoic mass on the rectal wall that penetrated the submucosa and muscularis propria and infiltrated the perirectal tissue (arrow); the diagnosis was T3 rectal cancer. (**B**) An MFI mode image revealed that the microblood flow signals of the mass were abundant; there were more than four blood flow streams that had spread to more than 50% of the maximum section of the tumor. (**C**) Magnetic resonance imaging revealed tumor signals encroaching the muscle layers and reaching the perirectal fat (arrow). The diagnosis was T3 rectal cancer. (**D**) The pathologic diagnosis was moderately differentiated adenocarcinoma stage T3 as ulcerative type; the cancerous tissue (long black arrow) penetrated the muscularis propria (short black arrow) and invaded the subserosa (white arrow) and nerve

**Fig. 5:**
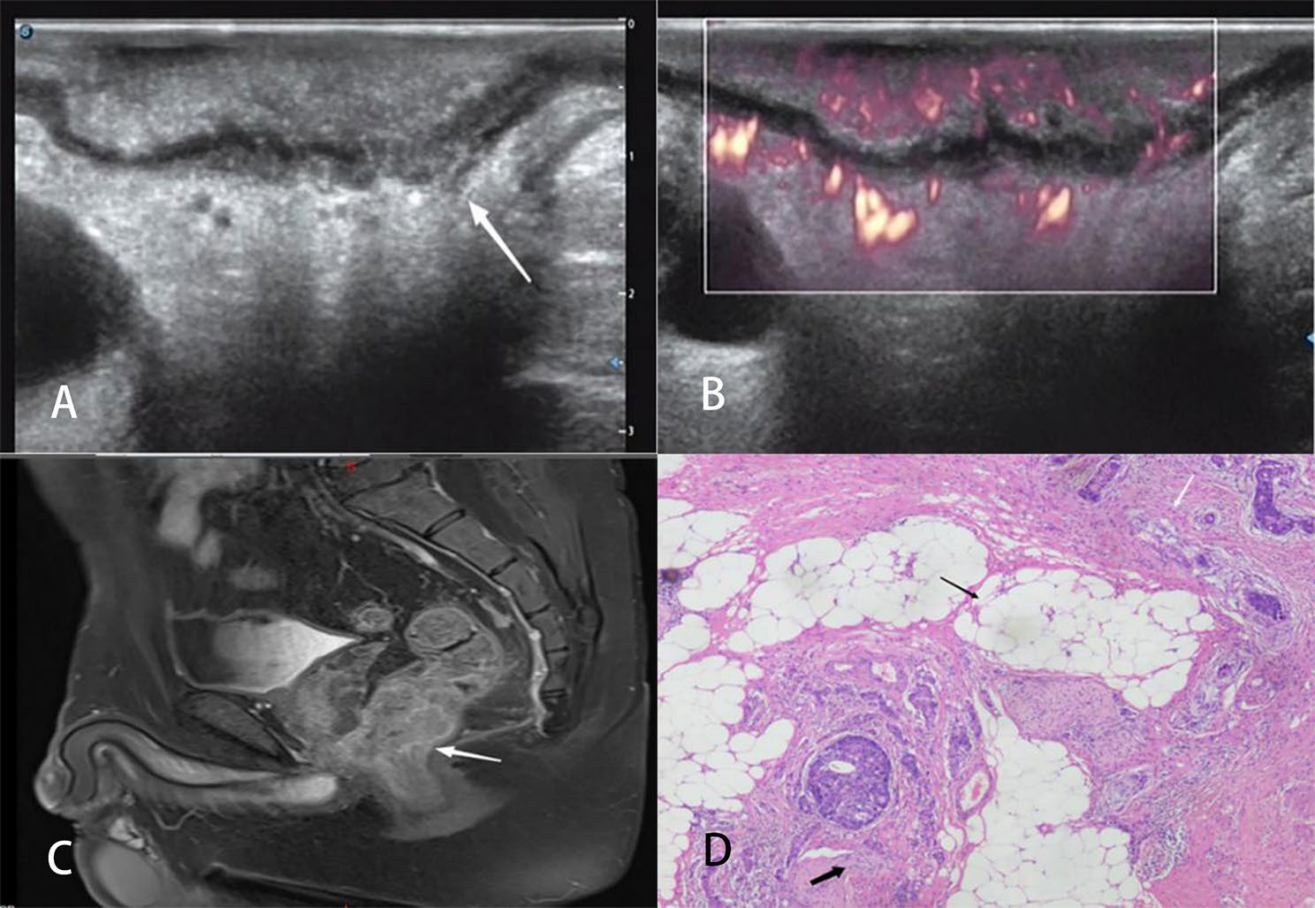
Images of a 42-year-old man with a T4 stage rectal tumor. (**A**) Two-dimensional endorectal biplane ultrasound of the line array revealed a hypoechoic mass on the rectal wall that invaded the adjacent organs and nearby pelvic tissues (arrow). The diagnosis was T4 rectal cancer. (**B**) MFI mode image showing that the microblood flow signals of the mass are relatively abundant, with 3–4 short streams distributed below 50% of the maximum section of the tumor. (**C**) Magnetic resonance imaging revealed thickening of the wall of the lower rectum under peritoneal reflection (arrow); the diagnosis was T4 rectal cancer. (**D**) The pathologic diagnosis was moderately differentiated adenocarcinoma stage T4 as ulcerative type; the cancerous tissue invaded the adjacent organs and pelvic tissues (white long arrow, serosa; black long arrow, perirectal fat; black short arrow, nerve)

Ultrasonic T classification for rectal cancer was performed by reviewing two-dimensional ultrasonography, color Doppler ultrasound images, and MFI data and following the staging criteria proposed by Hildebrandt and Feifel (1985) [18]. The specific definitions are as follows: stage uT1, tumor invasion is confined to the mucosa or submucosa, with a continuous hyper-echoic submucosa (Fig. 2); stage uT2, the tumor has penetrated the submucosa and invaded the muscularis propria, but is localized in the rectal wall (Fig. 3); stage uT3, the tumor has infiltrated the perirectal tissue without peritoneal coverage (Fig. 4); and stage uT4, the tumor has invaded the adjacent organs or nearby pelvic tissues (Fig. 5).

**Fig. 6:**
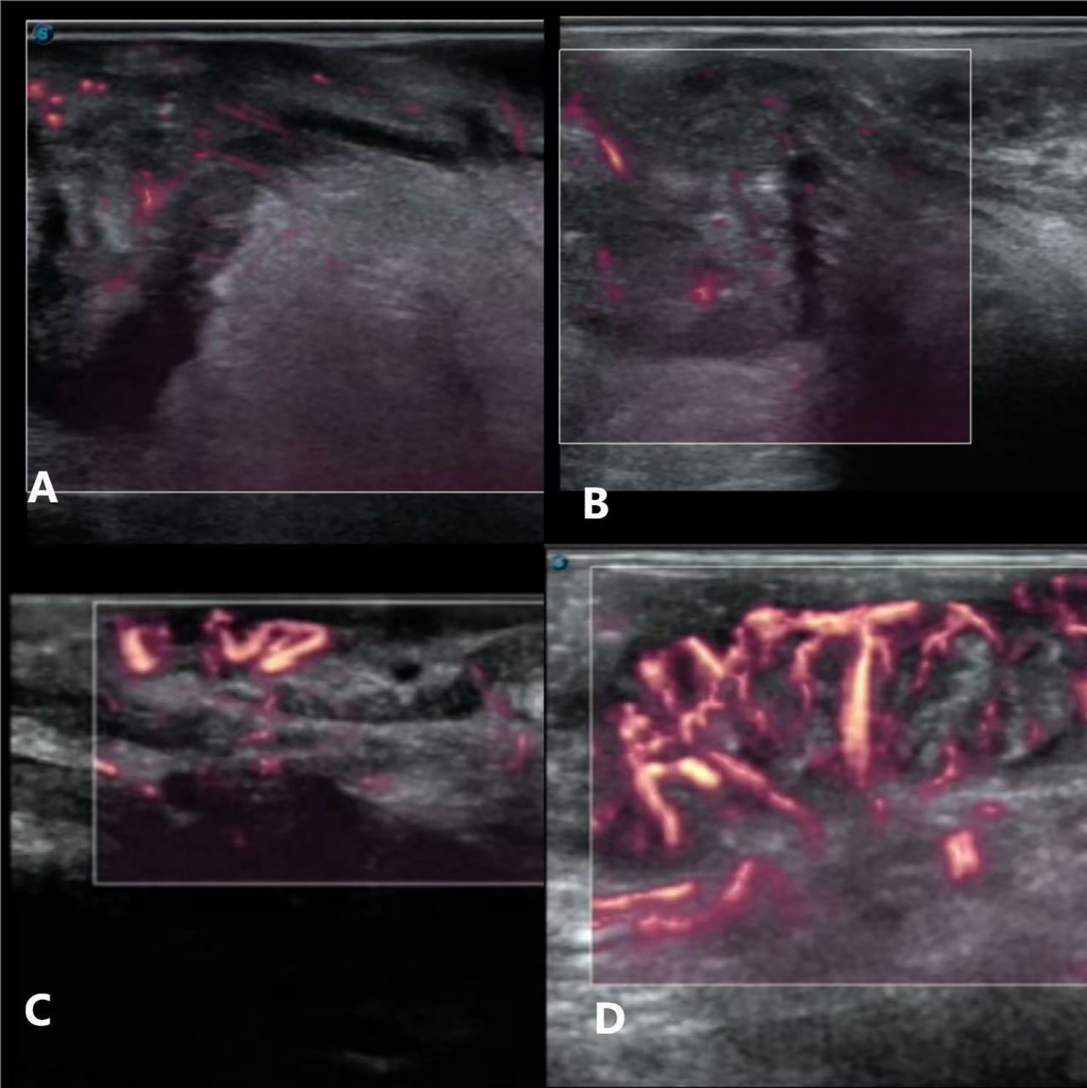
Four MFI grades of RC.

MFI classification was determined using the Adler semi-quantitative method [16]: grade 0: no microscopic flow in the tumor (Fig. 6A); grade 1, 1–2 dotted flow signals in the sparse tumor (Fig. 6B); grade 2, 3–4 short streams distributed below 50% of the maximum section of the tumor (Fig. 6C); and grade 3, more than four blood flow streams and spread more than 50% of the maximum section of the tumor (Fig. 6D).

MRI staging was determined as follows: T1: tumor signals were detected in adjacent submucosa, and did not exceed the submucosa; T2: tumor signals had invaded the muscle layers, the frontier of the muscle layers, and the submucosa had disappeared; T3: tumor signals encroached the muscle layers and reached the perirectal fat; T4: tumor signals had invaded the surrounding structures or organs.

### Statistical analysis

Data were analyzed using SPSS 26.0 statistical software (IBM, Armonk, NY, USA). Data were expressed as means ± standard deviation (means ± SD) and compared using the t-test. MFI grades were compared with postoperative pathological T stages using the rank-sum test. An independent samples t-test was used to compare the index differences between the two groups, and the chi-square test was used to compare the index differences among groups.

The kappa statistic was used to analyze the agreement of the classifications of TRUS, MRI with postoperative pathological T stages of rectal cancer using Cohen’s kappa (*K*) coefficient. *K* ≤ 0.40 indicated poor consistency; 0.40 < *K* ≤ 0.60 indicated moderate consistency; 0.60 < *K* ≤ 0.80 indicated high consistency; and *K* > 0.80 indicated excellent consistency. Statistical significance was set at *p* < 0.05.

The receiver operating characteristic (ROC) curve were performed to evaluate the diagnostic effectiveness of the CEA and CA199.

## Results

### Postoperative pathological results

This study included 87 patients with rectal cancer. The tumor tissues were evaluated by pathological examination following the 8th AJCC staging system. The 87 tumors included 23 (26.44%) cases of pT1, 17 (19.54%) cases of pT2, 30 (34.48%) cases of pT3, and 17 (19.54%) cases of pT4 (Table 1).

### MFI grades of rectal cancer

MFI grades of tumors were evaluated using the Adler semi-quantitative method. There were no cases of grade 0, 22 cases of grade 1, 38 cases of grade 2, and 27 cases of grade 3 (Table 1). There were no significant correlation between Adler classification and pathological T staging (*p* = 0.648) (Table 1).

**Table 1:** The MFI grades and pathological T stages of rectal cancers

MFI-grade	Pathological T stage				Total	*P* value
	pT1	pT2	pT3	pT4		
1	6	6	4	6	22	0.648
2	8	7	16	7	38	
3	8	4	9	6	27	
Total	23	17	30	17	87	

### Results of biplane TRUS combined with MFI T staging of rectal cancer

Two-dimensional sonography of the rectal cancer mass showed that the mean distance from the inferior margin to the anal verge was 5.2 ± 2.2 cm. From color Doppler, the mean RI was 0.70 ± 0.11. Ultrasonic T staging results were as follows: 19 (21.83%) cases of uT1, 15 (20.69%) cases of uT2, 42 (48.28%) cases of uT3, and 11 (12.64%) cases of uT4. Among the 87 patients, 63 (72.4%) were diagnosed with correct T staging, and the Cohen’s kappa coefficient K value was 0.615 (Table 2); 24 (27.6%) were diagnosed with incorrect T staging. Of the 24 mis-staged patients, 14 (58.3%) were overstaged, including 4 patients with stage T1 misclassified as stage T2, 2 patients with stage T1 misclassified as stage T3, 7 patients with stage T2 misclassified as stage T3, and 1 patient with stage T3 misclassified as stage T4. The other 10 patients (41.7%) were understaged, including 1 patient with stage T2 misclassified as stage T1, 2 patients with stage T3 misclassified as stage T2, 6 patients with stage T4 misclassified as stage T3, and 1 patient with stage T4 misclassified as stage T1. The sensitivity, specificity, and positive and negative predictive values for each stage (uT1–uT4) are listed in Table 3.

**Table 2:** Comparison of rectal cancer T staging results by TRUS with MFI and pathologic T staging

Ultrasonic T stage	Pathological T stage				total(n)	%	Ultrasonic T stage [(n/%)] of patients		
	pT1	pT2	pT3	pT4			Over-staged	Under-staged	Correctly staged
uT1	17	1	0	1	19	73.9	6(26.09)	0(0.00)	17(73.91)
uT2	4	9	2	0	15	52.9	7(41.18)	1(5.88)	9(39.13)
uT3	2	7	27	6	42	90.0	1(3.33)	2(6.67)	27(90.00)
uT4	0	0	1	10	11	58.8	0(0.00)	7(41.18)	10(58.82)
Total	23	17	30	17	87		14(16.09)	10(14.49)	63(72.41)

*K =* 0.615, *p* < 0.001

**Table 3:** Sensitivity, specificity, positive and negative predictive values for rectal cancer T staging by biplane TRUS plus MFI

Ultrasonic T stage	Sensitivity%	Specificity%%%	Positive predictive% value%	Negative predictive value%
uT1	73.9(17/23)	96.9(62/64)	89.5(17/19)	91.2(62/68)
uT2	52.9(9/17)	91.4(64/70)	60.0(9/15)	88.9(64/72)
uT3	90.0(27/30)	73.7(42/57)	64.3(27/42)	93.3(42/45)
uT4	58.8(10/17)	98.6(69/70)	90.9(10/11)	90.8(69/76)

The kappa value indicated that the performance of biplane TRUS plus MFI in the rectal cancer preoperative T staging was highly consistent with pathological T staging (*K =* 0.615, *p* < 0.001)

### Comparison of TRUS, MRI and CEA/CA199 in T staging of rectal cancer

Among the 87 patients with rectal cancer, 64 underwent MRI examinations and serum CEA and CA199 evaluation. The preoperative T stagings of the 64 rectal cancer patients diagnosed by TRUS and MRI data were shown in Table 4a–4b. The detection rates of TRUS and MRI were 71.88% (*K =* 0.599, *p <* 0.001), and 51.56% (*K =* 0.303, *p <* 0.001), respectively. MRI showed sensitivity and specificity rates between 23.1 and 79.2% and 55.0–100%, while TRUS showed rates of 53.8–95.8% and 70–100% (Table 5). TRUS enabled accurate staging at each T stage and TRUS led to higher accuracy of diagnosis compared with MRI .

**Table 4a:** Comparison of rectal cancer T staging by TRUS (uT staging) and pathology (pT staging)

Ultrasonic T stage	Pathological T stage				Total(n)
	pT1	pT2	pT3	pT4	
uT1	7	0	0	1	8
uT2	4	7	1	0	12
uT3	2	6	23	4	35
uT4	0	0	0	9	9
Total	13	13	24	14	64

*K =* 0.599, *p* < 0.001

**Table 4b:** Comparison of rectal cancer T staging by TRUS (uT staging) and pathology (pT staging)

Ultrasonic T stage	Pathological T stage				Total(n)
	pT1	pT2	pT3	pT4	
uT1	7	0	0	1	8
uT2	4	7	1	0	12
uT3	2	6	23	4	35
uT4	0	0	0	9	9
Total	13	13	24	14	64

*K =* 0.599, *p* < 0.001

**Table 5:** Comparison of the diagnostic performance of TRUS and MRI for each tumor stage

Stage	Ultrasonic T stage					MRI staging				
	N	SE	SP	PPV	VPN	N	SE	SP	PPV	VPN
T1	8	53.8	98. 0	87.5	89.3	3	23.1	100	100	83.6
T2	12	53.8	90.2	58.3	88.5	12	30.8	84.3	18.2	82.7
T3	35	95.8	70.0	65.7	96.6	37	79.2	55. 0	51.4	81.5
T4	9	64.3	100	100	90.9	12	50.0	90.0	58.3	86.5

**Fig. 7:**
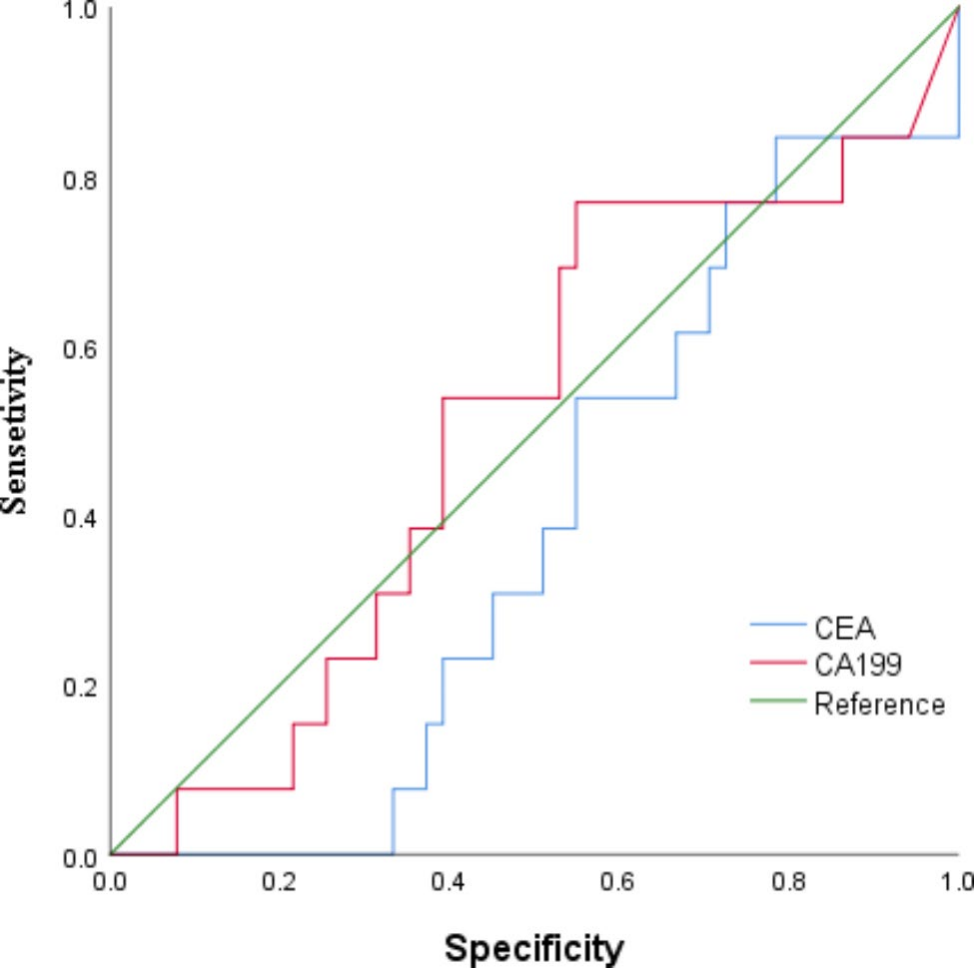
ROC curve of CEA and CA199. The AUC of CEA is 0.382; the AUC of CA199 is 0.507

The serum CEA and CA199 levels in patients with different pathological stages were different, and the positivity rate of serum CEA and CA199 increased with stage (*p* < 0.001; Table 6). Independent-samples t-test was used to compare index differences between the two groups. The area under the curve (AUC) of CEA and CA199 was 0.382 and 0.507, respectively (Fig. 7).

**Table 6:** The serum CEA and CA199 ($\stackrel{-}{\text{x}}$± s) levels of rectal cancer patients with different pathological stages

pT stage	n	CEA	CA199
pT1	13	2.64 ± 1.43	6.61 ± 2.30
pT2	13	3.30 ± 1.36	15.66 ± 5.70
pT3	24	10.21 ± 5.44	32.69 ± 15.49
pT4	14	21.69 ± 12.10	47.45 ± 15.34

**Fig. 8:**
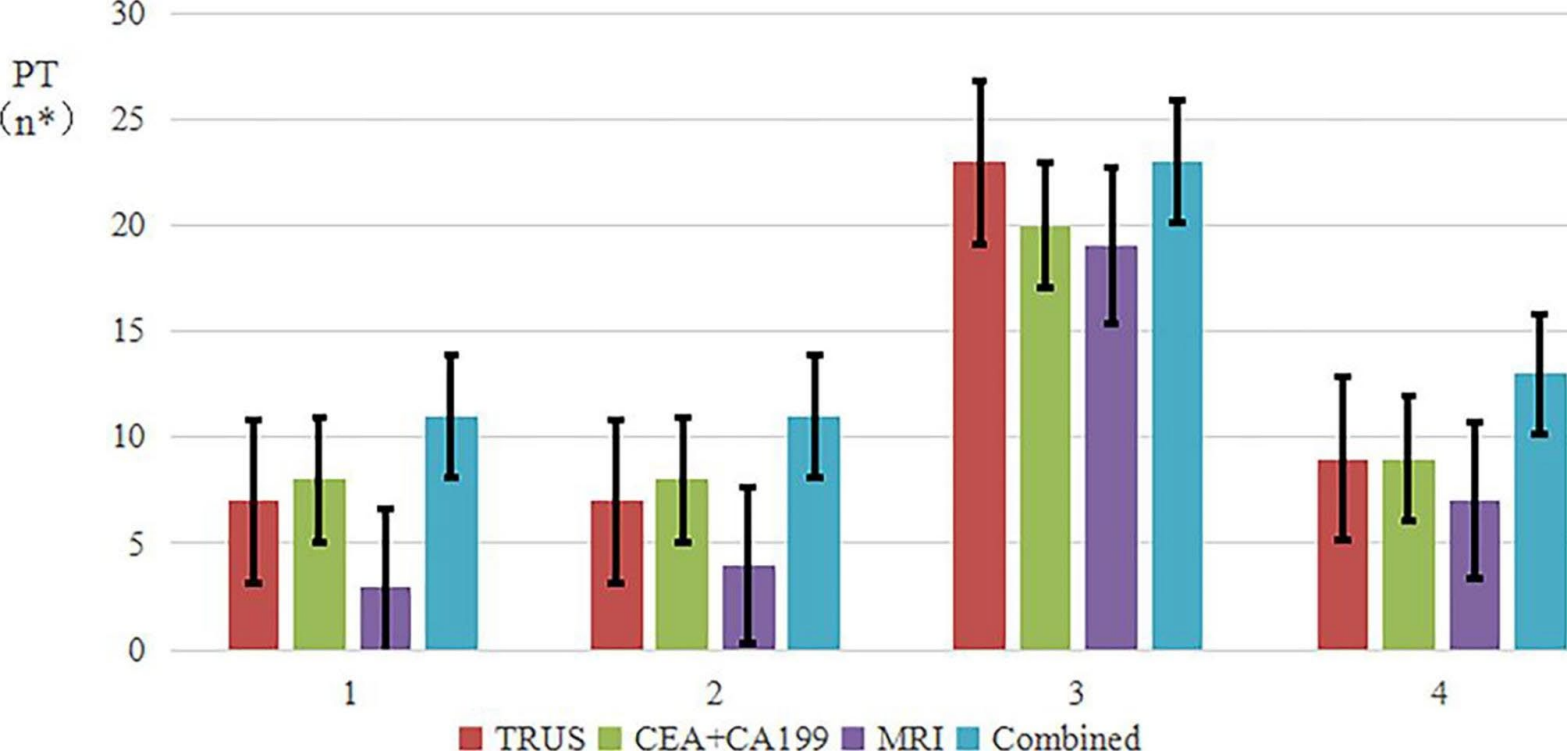
Accuracy of the four examination methods. n*, number of patients

With the combined detection of CEA/CA199 levels, MRI, and TRUS, the diagnostic concordance rate was higher than that with only one detection method, and the combined evaluation allowed a more accurate judgment of preoperative T staging (Table 7). This was especially true in stages T2 and T4, which were not easily diagnosed (Fig. 8).

**Table 7:** Comparison of rectal cancer T staging results among the four examination methods

Examination method	pT staging				Accuracy (%)
	pT1	pT2	pT3	pT4	
PT	13	13	24	14	100.0
TRUS	7	7	23	9	71.9
CEA + CA199	8	8	20	9	70.3
MRI	3	4	19	7	51.6
Combined	11	11	23	13	90.6

## Discussion

Intraluminal ultrasound was first introduced 38 years ago [18]. As a noninvasive examination method, this modality is safe and easy to operate, with low costs, and is a dynamic modality that provides real-time data [19]. Intraluminal ultrasound can identify the wall structure of the intestine and delineate the five layers of the rectal wall into three hyperechoic layers and two hypoechoic bands [20]. With these advantages, this technique has become an indispensable examination method for the preoperative T-staging of rectal cancer. Chan et al. showed that TRUS has better performance in diagnosing T1 and T3 rectal tumors than MRI [21], which is in agreement with our experimental results. The current diagnosis and treatment standard recommends TRUS as the conventional choice for the diagnosis of preoperative T staging of middle and low rectal cancer [22].

TRUS was used to perform preoperative T-staging of rectal cancer patients by observing the depth of mass infiltration in the intestinal wall [23]. The biplane transrectal ultrasonic probe has two beam emission modes, a convex array (fan-shaped image) and a line array (rectangular image), which show the cross-section and longitudinal section of the rectum, respectively. The combination of these two modes would allow more comprehensive imaging. In our study, the accuracy rate with TRUS (72.4%) was not satisfactory compared with that of other studies, which reported accuracy rates as high as 94.8% [24].

MFI is a high-resolution blood flow imaging technique based on the Doppler principle that was developed in recent years and is similar to superb microvascular imaging (SMI). The MFI technique from Sonoscape Medical Corporation uses the Matrix E adaptive matrix filter, which can accurately evaluate microvessels and extremely low-speed blood flow without using the ultrasound contrast agent by filtering signals of soft tissue and noise and effectively removing the Doppler mixed wave. Research has shown that MFI technology reveals more blood vessels and clearer flow distribution in liver lesions than traditional color Doppler flow imaging (CDFI), and it can improve the accuracy of the diagnosis of focal liver lesions [25–27]. Zhu et al. found that MFI perfusion characteristics of gallbladder polyps were in good agreement with findings of contrast-enhanced ultrasound (CEUS), which provided an accurate differential diagnosis between neoplastic and non-neoplastic gallbladder polyps [28]. Research has also shown that the MFI technology is in high agreement with CEUS in displaying tiny blood vessels (K = 0.84) [29]. Lin et al. previously showed that MFI can evaluate micro blood flow in detail, and it can be used to assess the vascular features contributing to the diagnosis of malignant breast masses; this association may be modified by age [30]. MFI has a high predictive value for cytological malignancies in neck metastases by assessing peripheral vascularization [10]. Conversely, Cappelli et al. suggested that MFI shows tiny blood vessels in more detail [31]. Thus, the detection rate of thyroid cancer has improved. The application of MFI technology has achieved significant achievements in multiple diseases [32], but little research has been conducted on its ability of evaluating microvessels in rectal cancer. In our study group of 87 patients, the MFI scoring revealed no cases of grade zero, 22 cases with 1 point, and 65 cases with 2 or 3 points. While there were no significant differences between the Alder classification and pathological T staging, our results nevertheless showed that rectal tumors are rich in micro-blood flow and that the MFI technique can display very low-speed tiny vessels in all stages of rectal cancer and sensitively capture blood flow signals.

The combination of improved B-mode TRUS, Doppler, and MFI led to the development of multiparametric ultrasonography (mpUS), which accurately detects rectal cancer. Multimodal ultrasound has been applied in malignant tumors such as breast cancer, thyroid cancer, and liver cancer. However, few studies have examined its application in rectal cancer T staging.

In this study, among the 24 patients with inconsistent uT and surgical pathological stages, 14 (58.3%) were over-staged and 10 (41.7%) were understaged. Most misclassified cases were overstaged, and this may be caused by several factors. First, overstaging is mainly affected by the peritumoral tissue reaction. Inflammation around the tumor can occur unexpectedly after neoadjuvant chemoradiotherapy, resulting in blurred boundaries, and it is difficult to confirm where the tumor has invaded the muscle layer. For artifacts formed by inflammation, it is difficult to distinguish the surrounding inflammatory lesions from the masses. Thus, inflammation is the major and most common factor contributing to overstaging. Second, ulcers on the surface of some tumors form scars and fibrosis, which are hypoechoic and illegible on traditional biplane TRUS. Third, tumors close to the anal canal and transverse folds of the rectum tend to be mistaken for infiltration. Fourth, in rectal cancer with a narrow bowel lumen, the probe cannot pass, which limits the diagnosis. Finally, inflammatory sites are associated with abundant blood supply; therefore, neither MFI nor color Doppler flow imaging can clearly distinguish the boundaries, leading to excessive staging.

Several reasons may explain the understaging of the 10 patients with rectal cancer in this study. (i) Some tumors with minor invasive lesions are difficult to clearly distinguish by ultrasound. (ii) Large masses limit the range of motion of the probe, and the extent of invasion of the intestinal wall cannot be fully displayed. (iii) The location of the tumor may be too high to clearly distinguish the boundary between the intestinal wall and tumor, leading to incorrect assessments. (iv) Postoperative recurrence of rectal cancer causes unpredictable interference factors leading to understaging.

CEA and CA199 have been proven to be significant for the diagnosis and prognosis of rectal cancer [14, 33, 34]. CEA and CA199 have been recommended by the American Society of Clinical Oncology (ASCO) as prognostic biomarkers to determine the prognosis and stage of rectal cancer [35]. Serum CEA shows different positive rates in diagnosing colorectal cancer, pancreatic cancer, gastric cancer, liver cancer, and other malignant tumors. CA199 is a well-recognized and widely used marker for colorectal cancer. Our results showed that serum CEA and CA199 levels gradually increased with the increase in staging. The accuracy rate was 71.9%, indicating that CEA and CA199 levels have some reference value for preoperative T staging of rectal cancer, while preoperative T staging of RC diagnosed by CEA and CA199 was of poor consistency. CEA and CA199 is affected by nodal status and distant metastasis, while our research didn’t take into N or M stage, which may run out error. The accuracy rate was improved in the combination of CEA and CA199 with MRI and TRUS.

Since 1986, MRI has been used for the preoperative diagnosis of rectal cancer. MRI has an advantage in displaying the soft tissue, and it can distinguish each intestinal wall structure and the surrounding fat fascia and identify the pelvic lymph nodes and possible vascular infiltration, enabling accurate clinical staging of the disease [6, 36–38]. In our study, the accuracy of MRI diagnosis was 51.6%, which was lower than that of TRUS (71.9%). TRUS had sensitivity and specificity rates of 53.8–95.8% and 70–100% respectively, while those of MRI were 23.1–79.2% and 55.0–100%. TRUS showed more sensitivity in T2–4 compared with MRI, while MRI had a higher specificity in T1 than TRUS. The sensitivity and specificity ranges of preoperative MRI at 23.1–79.2% and 55.0–100.0%, respectively, were consistent with previous findings [39]. The misclassified cases may be caused by the following reasons. First, TRUS was shown to be superior in judging rectal cancer at an early stage (including T1 and T2 stages) from its advantages in identifying the submucosa and muscularis propria [36, 40–43]. Therefore, this may be related to the relatively large number of early stage cases we selected. Additionally, some studies have shown that a 3.0-T MRI improved the diagnostic accuracy of tumors at all stages, with an accuracy of 86–95%, compared with 1.5-T MRI [44–46]. In our study, we used both types, which may have influenced the results of preoperative T staging. Moreover, unlike the TRUS stage results, which were diagnosed by a single senior doctor and a single junior doctor, the MRI staging results were judged by several different doctors, resulting in inevitable personal bias.

Our study has some limitations. First, the sample size of this study was relatively small, partly because of the lack of postoperative pathological results and incomplete imaging data; therefore, a larger sample size is needed for further studies. Second, the diagnostic results depended on the examiner’s experience and technical ability; Therefore, the evaluation by radiologists with different levels of experience may have influenced consistency and accurate results. Third, patients with factors that affect CEA, such as smoking, hepatopathy, nephropathy and diabetes, were not excluded in our study, which may have caused a false-positive CEA increase [33]. Fourth, we did not include shear wave transrectal ultrasound, which might have improved the results [47].

## Conclusions

This study demonstrated that rectal cancer staging with biplane TRUS plus MFI is highly consistent with pathological findings. As a promising new technique, MFI has significant advantages in showing intralesion microflow. The consistency of TRUS and MFI in preoperative T-stage testing in rectal cancer outperformed MRI. Serum CEA and CA199 levels increased with tumor stage. The combined application of biomarkers CEA/CA199, TRUS, and MRI examination had a higher diagnostic rate than any examination alone and was able to overcome their respective limitations in recognising tissue infiltration of intestinal wall by providing complementary advantages, thus improving the accuracy of preoperative T staging of rectal cancer and providing more accurate data for the clinical diagnosis of rectal cancer. However, the sample size of this study was small, and the combined diagnosis of RC requires further investigation in larger patient groups.

## Data Availability

The data supporting the findings of this study are available from the corresponding author upon reasonable request.
